# Supplementary material for: Multidose Hyaluronidase Administration as an Optimal Procedure to Degrade Resilient Hyaluronic Acid Soft Tissue Fillers
Source: Molecules. 2023 Jan 19;28(3):1003. doi: 10.3390/molecules28031003 (PMC9919540; doi:10.3390/molecules28031003)
Supplement: Supplementary file 1 [file molecules-28-01003-s001.zip › molecules-2101345-supplementary.pdf]

Supplementary Materials

Supplementary methods

Method for the assessment of the HA chain degradation kinetics using size exclusion chromatography

The evaluation of HA degradation was based on the multidose rheological testing. 1 g of gel was introduced in a tube and incubated at 37°C. Every 5 min, 50 µL of HAase was incorporated in the gel under gentle stirring until complete gel degradation. After every timepoint, HAase was deactivated at 100 °C for 3 min before adding 10 mL of phosphate buffer for HA fragments extraction. After 3 days of extraction at 37 °C, the supernatant – containing HA fragments - was retrieved from the insoluble gel part and filtered at 0.45 µm (Minisart NML Plus, Sartorius). The mass average molar mass (or molecular weight, Mw) and the proportion of low molecular weight HA fragments released were quantified by HPLC-SEC (1260 Infinity II, Agilent) equipped with a multiangle laser light scattering detection (Dawn Neon MALS, Wyatt Technology Corp., USA) and a refractive index detection (Optilab dRI, Wyatt Technology Corp., USA). A dual set of size exclusion columns OHpak LB-806M held in series flow (Shodex, Japan) were used. The mobile phase (150 mM $NaNO_3$ including 200 ppm NaN_3) was used at a flow rate of 0.3 mL/min. Refractive index increment (dn/dc) was set at 0.165 mL/g. The ASTRA 8 software (Wyatt Technology Corp., USA) has been used to obtain and analyze the chromatograms.

Supplementary results

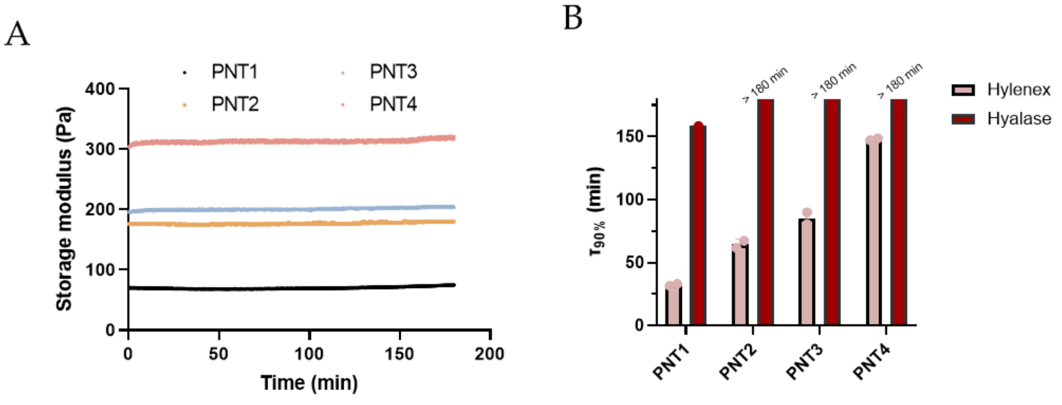

Figure S1. (A) Evolution of Teosyal RHA gels for 180 min without the addition of the enzymes, as followed by rheological time sweeps, (B) time to reach 90% gel degradation for PNT gels using the Hylenex or Hyalase enzyme.

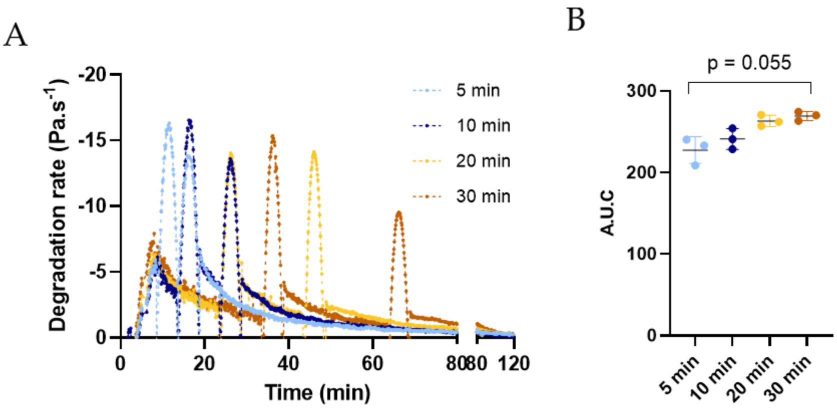

Figure S2. Degradation rates (A) and area under the curve (B) derived from PNT4 degradation curves with triple injections of total 25 U of Hylenex per mL of gel at different intervals of 5, 10, 20, and 30 minutes. Results on B are presented as mean ± S.D, n = 3.

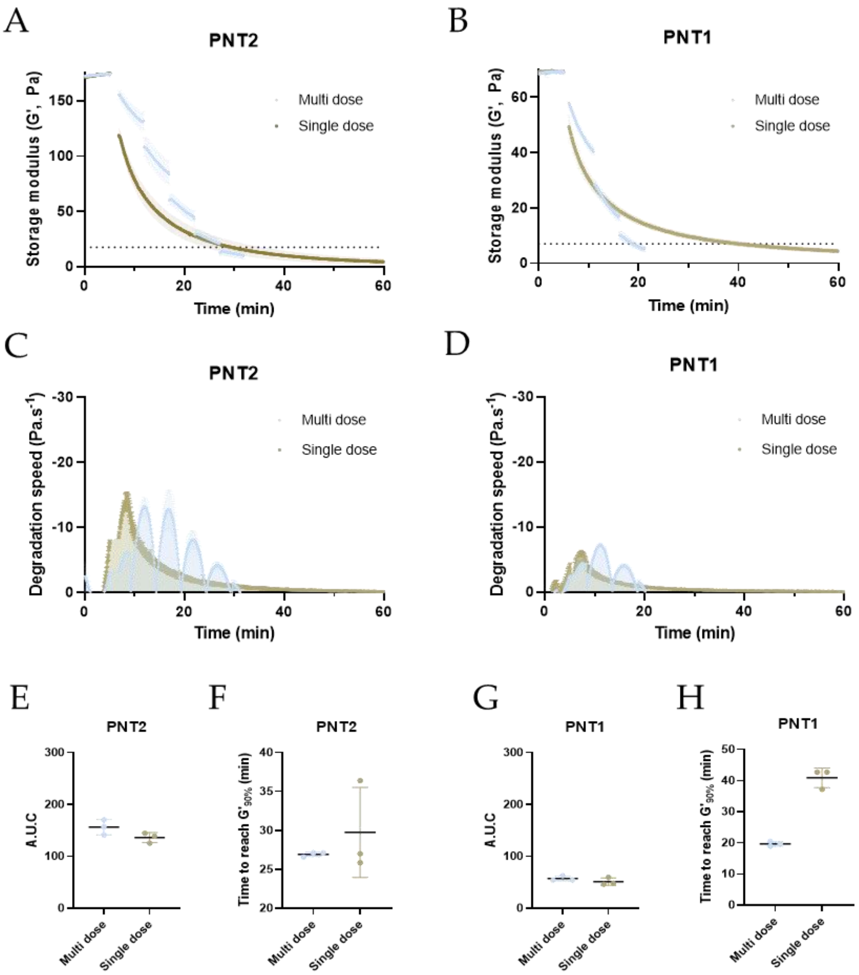

Figure S3. Optimization of PNT2 and PNT1 degradation parameters by multiple injections of Hylenex, followed by rheological time sweeps. Evolution of the storage modulus of PNT2 (A) and PNT1 (B) gels overtime, measured by time sweeps (37 °C, strain 0,1%, 1 Hz), following single or multiple injections of the Hylenex enzyme (38.3 U·mL^{-1} per injection, 5 min injection span for multidose), total volume and enzymatic units were equivalent for single and multidose. Dotted lines represent 10% of initial G′, area under the curve for PNT2 (E) and PNT1 (G) gels calculated

from the first derivative curves, time required to reach 90% of gel degradation for PNT2 (F) and PNT1 (H) gels, relative to the initial storage modulus. All results are presented as mean ± S.D, n = 3.

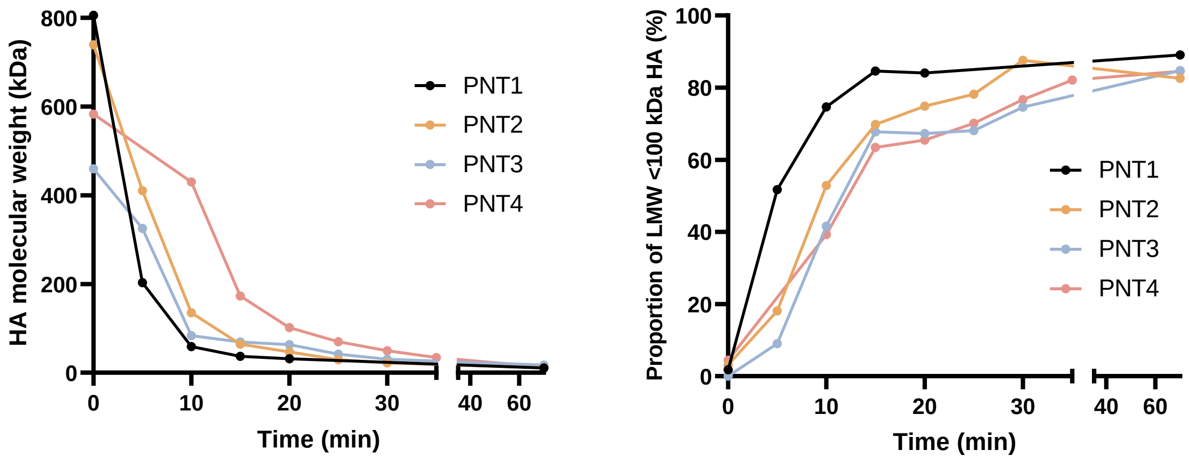

Figure S4. Monitoring of HA enzymatic degradation by HPLC-SEC showing the Mw of extractible sHA (left) and the proportion of LMw sHA < 100 kDa (right). Samples were taken from T0 to T0 + 70 min and analyzed directly after sample preparation.
